# Employment trends in schizophrenia and bipolar disorder in Spain: a nationwide register-based study (2018–2023)

**DOI:** 10.1007/s00406-026-02279-x

**Published:** 2026-06-04

**Authors:** Vicent Llorca-Bofí, Gerard Anmella, Daniel Guinart, Catalina Paredes-Coronel, Diego Hidalgo-Mazzei, Eduard Vieta, Miquel Bioque

**Affiliations:** 1 Barcelona Clínic Schizophrenia Unit (BCSU), Hospital Clínic de Barcelona, Neuroscience Institute, Institut d’Investigacions Biomèdiques August Pi I Sunyer (IDIBAPS), Barcelona, Catalonia Spain; 2 Department of Medicine, School of Medicine and Health Sciences, Institute of Neurosciences (UBNeuro), University of Barcelona (UB), Barcelona, Catalonia Spain; 3 Biomedical Research Networking Centre Consortium on Mental Health (CIBERSAM), Instituto de Salud Carlos III, Madrid, Spain; 4 Bipolar and Depressive Disorders Unit, Hospital Clinic, Institut d’Investigacions Biomèdiques August Pi I Sunyer (IDIBAPS), Barcelona, Catalonia Spain; 5 Institut de Salut Mental, Hospital del Mar, Barcelona, Spain; 6 Department of Psychiatry, The Donald and Barbara Zucker School of Medicine at Hofstra/Northwell, Hempstead, NY USA; 7 Clinical and Organizational Innovations Unit, Quirónsalud 4-H Network, Madrid, Spain; 8 Instituto de Investigación Sanitaria, Fundación Jiménez Díaz (IIS-FJD), Madrid, Spain

**Keywords:** Schizophrenia, Bipolar disorder, Employment, Population-based data, Longitudinal trends, Spain

## Abstract

**Background:**

Employment is a key determinant of recovery in severe mental illness, yet longitudinal evidence from Southern Europe is limited. We aimed to examine national trends in employment status among individuals with schizophrenia and bipolar disorder in Spain between 2018 and 2023.

**Methods:**

We conducted a register-based trend study using the Spanish Primary Care Clinical Database (BDCAP), including adults aged 20–64 years with schizophrenia (n = 139,594), bipolar disorder (n = 148,968), or other diagnoses (n = 25,953,622). Employment status was classified as employed, unemployed, economically inactive, disability pensions, or other statuses. Trends were analysed with Joinpoint regression, reporting Average Annual Percent Change (AAPC) with 95% CIs.

**Results:**

In 2023, 15.2% of people with schizophrenia and 38.3% with bipolar disorder were employed, versus 62.5% with other conditions. Disability pensions were received by 49.3% and 26.5%, compared with 6.3% in other diagnoses. Between 2018 and 2023, employment rose modestly in schizophrenia (AAPC =  + 2.2; 95% CI 0.8–3.6; p < 0.001) and bipolar disorder (+ 2.4; 95% CI 1.6–3.2; p < 0.001), while disability pensions declined (–5.5; 95% CI − 8.9 to − 2.1; p = 0.003; and − 7.2; 95% CI − 9.4 to − 5.1; p < 0.001). The “other statuses” category grew sharply (schizophrenia + 52.8; bipolar disorder + 35.4; both p < 0.001). Sex-stratified analyses showed parallel trends, except for economic inactivity, which declined more steeply among women (Δ trend =  + 3.1, p = 0.029; + 2.7, p = 0.031; + 3.2, p = 0.008).

**Conclusions:**

Employment outcomes remain poor for people with schizophrenia and bipolar disorder in Spain. Most improvements likely reflect administrative changes rather than genuine integration into work.

## Background

Schizophrenia and bipolar disorder are severe mental disorders characterised by functional impairment and social exclusion [1–3]. Among working-age adults, employment is one of the most affected areas [4]. While bipolar disorder may allow intermittent labour participation during periods of remission, schizophrenia is typically associated with more persistent cognitive and social dysfunction, resulting in long-term unemployment and reliance on disability benefits in most patients.

Population-based studies from Northern Europe consistently report low employment rates: around 10–20% for individuals with schizophrenia, and 30–50% for those with bipolar disorder [5–8]. Despite advances in early intervention and access to services, trends over time show modest or even declining improvements in employment outcomes [5, 9], with persistent barriers including stigma, discrimination, and limited functional recovery [10, 11]. Sex disparities have also been described: women with severe mental illness tend to achieve slightly higher employment rates than men, yet face additional barriers such as caregiving responsibilities and labour market discrimination [6, 12]. However, most available evidence comes from Northern Europe, while data from Southern Europe—and particularly Spain—remain limited and insufficiently updated [13, 14].

Spain represents a relevant setting to address this gap. Its National Health System provides tax-funded, universal healthcare, with mental health services included in the public benefits package [15]. A strong primary care network enables the use of structured health records to track population-level trajectories [16]. In addition, Spain maintains a comprehensive social protection system, covering pensions, unemployment support, and disability benefits [17]. Legal reforms have also shaped the landscape: in 2021, Law 8/2021 replaced the concept of incapacitation with a rights-based model of supported decision-making [18]. Although not intended to alter labour classifications, this reform may have influenced the way individuals with long-term mental illness are administratively categorised.

Given the societal and economic burden of schizophrenia and bipolar disorder [19, 20], the structural importance of employment, and the context of recent legal and social changes in Spain, updated population-based evidence is urgently needed to inform mental health policy and service planning. To date, no study has examined national employment trends in these diagnostic groups using administrative health records.

To strengthen the evidence base, we analysed nationwide administrative health records to examine employment trends between 2018 and 2023 among individuals with schizophrenia and bipolar disorder. We compared these groups with individuals diagnosed with other health conditions, focusing on the distribution of labour categories by sex.

## Methods

### Study design

This is a register-based trend study analysing employment status in a representative sample of patients with schizophrenia, bipolar disorders and other health conditions in Spain for the period 2018–2023. The study design and reporting follow the STROBE (Strengthening the Reporting of Observational Studies in Epidemiology) guidelines [21].

### Data source

We used data from the Primary Care Clinical Database (BDCAP), an anonymised administrative registry maintained by the Spanish Ministry of Health. BDCAP was developed for research and statistical purposes with the collaboration of all autonomous communities in Spain. It compiles standardised clinical and sociodemographic information annually from electronic health records in the public primary care system. Although BDCAP has provided data since 2012, we restricted our analysis to 2018 onwards, when complete and consistent national coverage was first achieved.

The database uses a stratified single-stage cluster sampling design, based on Basic Health Areas, with strata defined by autonomous community and municipality size. Population weights are applied to ensure representativeness at both national and regional levels. Since the BDCAP does not include individual-level identifiers across years, individuals cannot be tracked over time; each annual extract represents an independent cross-section of the population registered within the sampled Basic Health Areas. Health conditions are coded by the primary care physician overseeing each Basic Health Area, in accordance with the International Classification of Primary Care, 2nd edition (ICPC-2), and the dataset includes data on diagnoses, prescribed and dispensed medications, demographic variables, and labour status. All records are pseudonymised and approved for secondary use in epidemiological research. This database has previously been employed in epidemiological and health services research in Spain [22–24]. Full methodological details are available at: https://www.sanidad.gob.es/estadEstudios/estadisticas/estadisticas/estMinisterio/SIAP/home.htm.

### Participants

Three cohorts were constructed from the database: Schizophrenia group (ICPC-2 code P72) bipolar disorder group (ICPC-2 code P73) and other health conditions (all remaining ICPC-2 codes). We included individuals aged 20 to 64 years. In Spain, individuals may enter the labour force from the age of 16 with parental or guardian authorization, whereas full legal capacity to work is granted at 18 years. However, BDCAP reports age in five-year bands, and individuals younger than 20 years could not be disaggregated. Therefore, only patients aged 20 years or older were included.

### Variables

The variables extracted for analysis included sex, age group, diagnostic category and employment status. Sex was recorded as male or female, while age was grouped in five-year intervals (e.g., 20–24, 25–29, etc.) as reported in the BDCAP dataset. Diagnoses were identified using the International Classification of Primary Care, version 2 (ICPC-2), as described in the previous section.

Employment status, as reported in the BDCAP dataset, was classified into five categories based on administrative health insurance affiliation and corresponded to the individual’s situation as recorded on December 31st of each study year, according to their affiliation status in the national social security system. These categories were: (1) *Employed*—individuals with active employment status and working rights; (2) *Unemployed*—individuals actively seeking work or receiving unemployment benefits; (3) *Disability pensions*—individuals receiving permanent disability or incapacity benefits; (4) *Economically inactive*—dependents or non-working individuals not included in the above categories (e.g., caregivers, adult students, household dependents); and (5) *Other statuses*—individuals affiliated through non-standard channels, such as special mutual insurance schemes or undefined categories.

### Ethical considerations

This study was conducted in accordance with the Declaration of Helsinki, and the Spanish Law 14/2007 on Biomedical Research [25], which exempts the need for ethics committee approval or informed consent in studies using anonymized, aggregated data. The BDCAP database contains no personal identifiers. Therefore, no ethical approval or patient consent was required.

### Statistical analysis

Data on the number of patients in each diagnostic and employment status group were extracted from the BDCAP using the filter “individuals with a health condition”. For each diagnostic group, sex, and year stratum, the percentage (%) distribution of employment status was calculated as the proportion of individuals in each category relative to the corresponding total.

Temporal trends were analysed using Joinpoint regression models [26, 27], which identify statistically significant changes in linear trends over time. Given the limited number of time points (2018–2023), only the Average Annual Percent Change (AAPC) was estimated for the entire study period, without fitting segmented trends. Model selection was guided by the Weighted Bayesian Information Criterion (WBIC) to minimise overfitting. Statistical significance was defined as *p* < 0.05, corresponding to 95% confidence intervals for the AAPC excluding zero.

To assess potential sex-differences in temporal trends, we calculated the Δ trend = AAPC(male) − AAPC(female), with the standard error estimated as SE ~ Δ ~  = √(SE^2^ ~ male ~  + SE^2^ ~ female ~), and statistical significance assessed using a two-tailed z-test (z = Δ trend / SE ~ Δ ~).

All data processing and visualisation were conducted in RStudio (v4.2.2), and Joinpoint analyses were performed with the Joinpoint Regression Program (U.S. National Cancer Institute).

## Results

### Sample characteristics

In 2023, the analytical sample included 139,594 individuals with schizophrenia (0.5%), 148,968 with bipolar disorder (0.6%), and 25,953,622 with other diagnoses (98.9%). The schizophrenia group was predominantly male (66.8%), whereas bipolar disorder was more frequent in females (57.4%). The age distribution showed that most patients with schizophrenia (65.3%) and bipolar disorder (59.4%) were aged 40–59. Table 1 shows the sociodemographic and employment characteristics of the sample in 2023.

**Table 1:** Sample characteristics by diagnosis, 2023

	Schizophrenia (n = 139,594)	Bipolar Disorder (n = 148,968)	Other Diagnoses (n = 25,953,622)
*Sex*			
Female	46,383 (33.2)	85,554 (57.4)	13,351,558 (51.4)
Male	93,211 (66.8)	63,414 (42.5)	12,602,064 (48.6)
*Age*, n (%)			
20–29	8,419 (6.1)	16,018 (10.7)	4,337,511 (16.7)
30–39	20,217 (14.4)	21,419 (14.3)	5,089,952 (19.6)
40–49	41,620 (29.8)	39,114 (26.2)	6,869,772 (26.5)
50–59	49,341 (35.5)	49,475 (33.2)	6,752,091 (26.0)
60–64	19,997 (14.3)	22,942 (15.4)	2,904,296 (11.2)
*Employment categories*, n (%)			
Employed	21,180 (15.2)	57,068 (38.3)	16,218,508 (62.5)
Unemployed	9,913 (7.1)	17,481 (11.7)	2,536,996 (9.8)
Economically inactive	16,520 (11.8)	17,570 (11.8)	3,024,328 (11.7)
Disability pensions	68,755 (49.3)	39,416 (26.5)	1,646,981 (6.3)
Other statuses	23,226 (16.6)	17,433 (11.7)	2,526,809 (9.7)

Values are presented as absolute frequencies and percentages (%). Percentages refer to the column total within each diagnostic group

### Employment status distribution in 2023

Marked differences in employment status were observed across diagnostic groups in 2023. The proportion of employed individuals was substantially lower in schizophrenia (15.2%) and bipolar disorder (38.3%) compared to other diagnoses (62.5%). Disability pensions were reported in 49.3% of individuals with schizophrenia and 26.5% with bipolar disorder, versus 6.3% of the rest of the population. Other employment categories showed smaller variations.

When stratified by sex, employment-related differences were minimal within diagnostic groups. Among individuals with schizophrenia, the employment rate was 15.0% in men and 15.5% in women; similarly, in bipolar disorder, rates were 38.7% and 38.0%, respectively—indicating no meaningful sex disparity in either group. Conversely, the general population exhibited a more marked sex gap, with 66.6% of men employed compared to 58.6% of women.

### Trends in employment status by diagnosis (2018–2023)

#### Schizophrenia

**Fig. 1:**
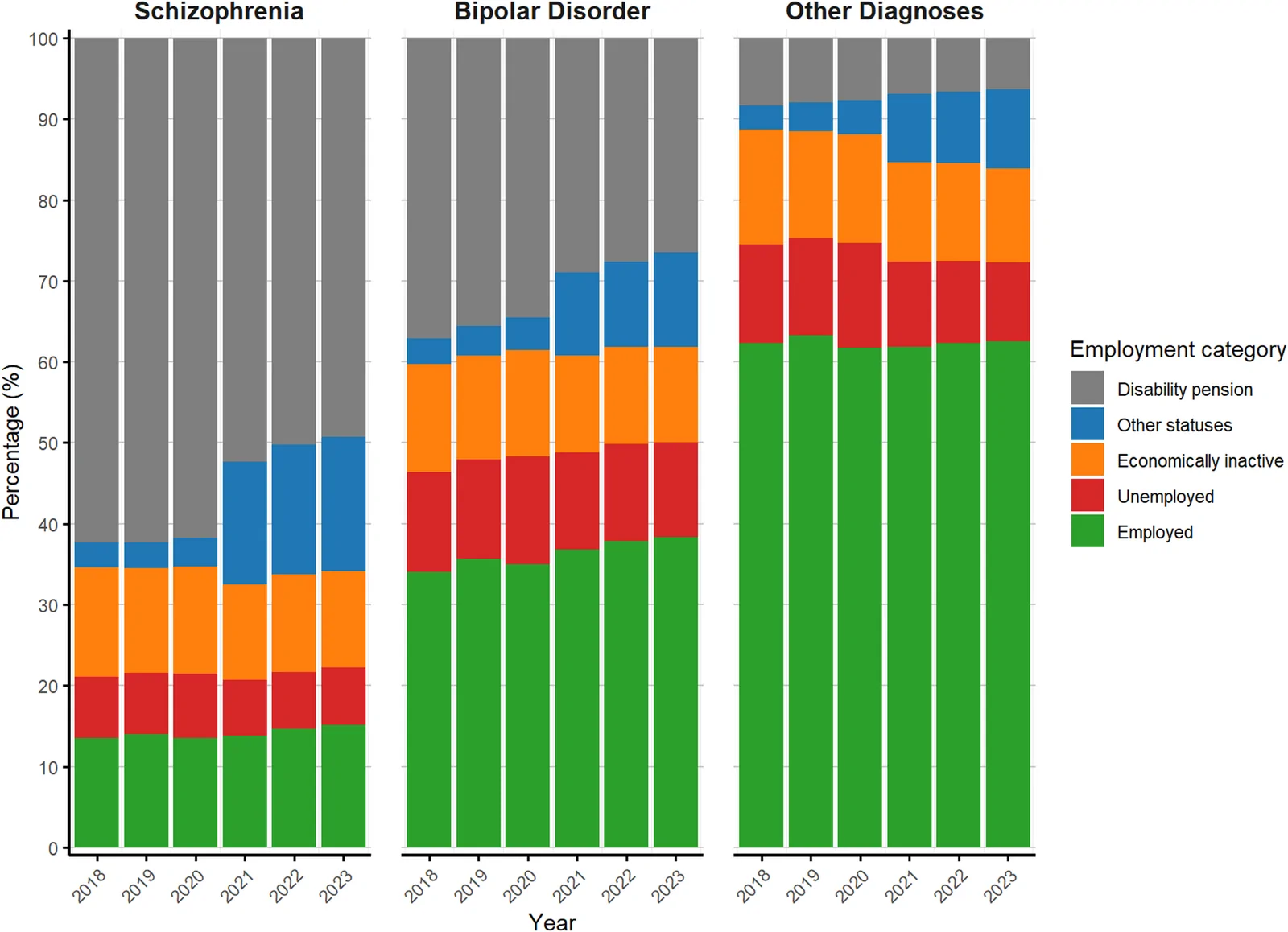
Trends in employment statuses by diagnosis (2018–2023). Stacked bar chart showing the annual distribution (%) of employment categories among individuals with schizophrenia, bipolar disorder, and other diagnoses between 2018 and 2023. Categories include: *employed* (active workers), *unemployed* (seeking work or receiving unemployment benefits), *economically inactive* (non-working individuals not actively seeking work. e.g. students. caregivers), *disability pensions* (recipients of permanent disability benefits), and *other statuses* (affiliations through special schemes or undefined administrative categories)

Between 2018 and 2023, individuals with schizophrenia showed modest but statistically significant improvements in employment, rising from 13.5 to 15.2% (AAPC =  + 2.2; 95% CI 0.8 to 3.6; p < 0.001). Disability pensions declined markedly from 62.3% to 49.3% (AAPC =  − 5.5; 95% CI − 8.9 to − 2.1; p = 0.003), while the “other statuses” category increased substantially (from 3.1% to 16.6%; AAPC =  + 52.8; 95% CI 18.2 to 96.3; p < 0.001). A significant decrease was also observed in the economically inactive category. Full results are presented in Table 2 and Fig. 1.

**Table 2:** Joinpoint analysis of employment status by diagnosis (2018–2023)

Diagnosis and employment categories	Percentages (%)		2018–2023	
	2018	2023	AAPC	p-value
*Schizophrenia*				
Employed	13.5	15.2	2.2 (0.8 to 3.6)*	< 0.001
Unemployed	7.6	7.1	− 2.1 (− 4.8 to 0.6)	0.134
Economically inactive	13.5	11.8	− 2.8 (− 4.3 to − 1.2)*	< 0.001
Disability pensions	62.3	49.3	− 5.5 (− 8.9 to − 2.1)*	0.003
Other statuses	3.1	16.6	52.8 (18.2 to 96.3)*	< 0.001
*Bipolar disorder*				
Employed	34.1	38.3	2.4 (1.6 to 3.2)*	< 0.001
Unemployed	12.3	11.7	− 1.2 (− 3.1 to 0.6)	0.191
Economically inactive	13.3	11.8	− 2.6 (− 4.1 to -1.2)*	< 0.001
Disability pensions	37.0	26.5	− 7.2 (− 9.4 to − 5.1)*	< 0.001
Other statuses	3.2	11.7	35.4 (17.2 to 55.7)*	< 0.001
*Other diagnoses*				
Employed	62.2	62.5	− 0.1 (− 0.8 to 0.6)	0.775
Unemployed	12.2	9.8	− 5.1 (− 8.1 to − 2.1)*	< 0.001
Economically inactive	14.3	11.7	− 3.8 (− 5.6 to − 1.9)*	< 0.001
Disability pensions	8.3	6.3	− 5.5 (− 6.4 to − 4.7)*	< 0.001
Other statuses	2.9	9.7	30.5 (17.3 to 45.1)*	< 0.001

*AAPC* Average Annual Percent Change

An asterisk (*) indicates statistical significance (p < 0.05)

#### Bipolar disorder

Employment rates increased from 34.1 to 38.3% (AAPC =  + 2.4; 95% CI 1.6 to 3.2; p < 0.001). Disability pensions declined sharply from 37.0% to 26.5% (AAPC =  − 7.2; 95% CI − 9.4 to − 5.1; p < 0.001), accompanied by a substantial increase in the “other statuses” category (from 3.2 to 11.7%; AAPC =  + 35.4; 95% CI 17.2 to 55.7; p < 0.001). Economically inactive individuals also declined significantly. Full results are available in Table 2 and Fig. 1.

#### Other diagnoses

Employment rates remained stable, changing only from 62.2 to 62.5%. Disability pensions decreased from 8.3 to 6.3% (AAPC =  − 5.5; 95% CI − 6.4 to − 4.7; p < 0.001), while the “other statuses” category rose from 2.9 to 9.7% (AAPC =  + 30.5; 95% CI 17.3 to 45.1; p < 0.001). Full results are presented in Table 2 and Fig. 1.

### Sex-stratified trends in age-adjusted rates

Sex differences in temporal trends were generally absent, with men and women showing parallel trajectories across most employment categories (Table 3). The only exception was economic inactivity, where women exhibited significantly steeper declines than men in all diagnostic groups (Δ trend =  + 3.1, p = 0.029 in schizophrenia; Δ trend =  + 2.7, p = 0.031 in bipolar disorder; and Δ trend =  + 3.2, p = 0.008 in other diagnoses).

**Table 3:** Joinpoint analysis of employment status and sex-differences in trends by diagnosis (2018–2023)

Diagnosis and employment categories	Male				Female				Sex-differences in trends	
	Percentages (%)		2018–2023		Percentages (%)		2018–2023		Δ trend (Men vs. Women)	p-value
	2018	2023	AAPC	p-value	2018	2023	AAPC	p-value		
*Schizophrenia*										
Employed	13.3	14.9	2.3 (0.4 to 4.1)*	0.011	13.7	15.5	2.1 (0.8 to 3.3)*	0.001	0.2	0.861
Unemployed	7.2	6.9	− 1.7 (− 4.6 to 1.3)	0.255	8.2	7.3	− 2.8 (− 6.9 to 1.3)	0.195	1.1	0.675
Economically inactive	11.8	11.1	− 1.5 (− 3.8 to 0.7)	0.178	16.8	13.1	− 4.6 (− 6.2 to − 2.9)*	< 0.001	3.1*	0.029
Disability pensions	64.9	50.9	− 5.6 (− 8.5 to − 2.7)*	< 0.001	57.3	45.8	− 5.4 (− 9.8 to − 0.9)*	0.020	− 0.2	0.944
Other statuses	2.6	15.9	56.7 (17.7 to 107.5)*	0.001	3.7	18.1	47.0 (19.2 to 80.4)*	< 0.001	9.7	0.738
*Bipolar disorder*										
Employed	34.9	38.7	2.3 (1.0 to 3.6)*	< 0.001	33.3	38.0	2.5 (1.2 to 3.7)*	< 0.001	− 0.2	0.830
Unemployed	11.0	10.8	− 1.1 (− 4.3 to 2.3)	0.452	13.2	12.3	− 1.4 (− 3.6 to 0.8)	0.225	0.3	0.881
Economically inactive	10.9	10.7	− 1.0 (− 4.7 to 2.9)	0.515	15.2	12.4	− 3.7 (− 5.6 to − 1.8)*	< 0.001	2.7*	0.031
Disability pensions	39.9	29.7	− 6.1 (− 7.5 to − 4.7)*	< 0.001	34.8	24.0	− 8.1 (− 11.4 to − 4.7)*	< 0.001	2.0	0.280
Other statuses	3.1	9.7	31.5 (12.5 to 52.7)*	< 0.001	3.2	13.1	37.8 (19.1 to 58.9)*	< 0.001	− 6.3	0.662
*Other diagnoses*										
Employed	67.3	66.6	− 0.3 (− 0.8 to 0.2)	0.222	57.6	58.5	0.1 (− 0.8 to 1.2)	0.673	− 0.4	0.483
Unemployed	10.5	8.7	− 4.4 (− 7.3 to − 1.3)*	0.004	13.7	10.7	− 5.6 (− 8.6 to − 2.6)*	< 0.001	1.2	0.579
Economically inactive	10.3	9.3	− 1.7 (− 3.3 to − 0.1)*	0.039	17.7	13.7	− 4.9 (− 6.6 to − 3.1)*	< 0.001	3.2*	0.008
Disability pensions	9.4	7.0	− 5.9 (− 6.8 to − 5.1)*	< 0.001	7.2	5.6	− 5.2 (− 6.1 to − 4.3)*	< 0.001	− 0.7	0.268
Other statuses	2.3	8.1	32.0 (18.9 to 46.2)*	< 0.001	3.5	11.2	29.6 (16.1 to 44.6)*	< 0.001	2.4	0.812

*AAPC* Average Annual Percent Change. Asterisks indicate statistical significance at p < 0.05: in AAPCs when the 95% confidence interval does not include 0; in Δ trend when the sex-difference test is significant. p-values in the last column correspond to the interaction test of differences in trends between men and women

## Discussion

This register-based trend study examined national trends in employment status among individuals with schizophrenia and bipolar disorder in Spain between 2018 and 2023. Our findings reveal four key patterns. First, employment rates remained substantially lower in individuals with schizophrenia and bipolar disorder compared with those diagnosed with other health conditions. Second, despite modest increases in employment, people with schizophrenia consistently showed the lowest levels throughout the study period. Third, marked declines in disability pensions coincided with substantial increases in the “other statuses” category, pointing to shifts in administrative classification. Finally, sex-stratified analyses showed that men and women followed broadly similar trends across most categories, with significant differences emerging only in economic inactivity, where women experienced steeper declines across all diagnostic groups. Together, these results underscore persistent barriers to employment for people with severe mental illness in Spain.

Our results are consistent with prior studies from Northern Europe reporting persistently low employment rates in schizophrenia, typically ranging between 10 and 20%, which are always lower than those observed in bipolar disorder [5, 6, 9]. In our study, both groups showed only modest improvements (AAPC + 2.2 for schizophrenia and + 2.4 for bipolar disorder), in contrast to individuals with other health conditions, whose rates remained essentially unchanged. This may reflect gradual advances in early intervention services, access to treatment, community support, or broader investments in public mental health infrastructure in Spain [28–30]. These changes may also partly mirror the general improvement in Spain’s labour market, where unemployment fell sharply between 2018 and 2023 [31] Nevertheless, progress remained limited, with employment reaching only 15% in schizophrenia and 38% in bipolar disorder by 2023, compared with 62% among individuals with other health conditions. This gap underscores the enduring functional burden of severe mental illness on labour participation and highlights that, beyond symptom improvement, functional recovery—particularly in terms of employment—remains a critical but often unmet objective [32, 33].

The reduction in disability pensions, particularly among those with schizophrenia, partially aligns with trends reported in other European settings [6, 9, 12]. Notably, these declines did not translate into parallel employment gains, which remained modest as previously mentioned. Instead, they were mirrored by substantial growth in the “other statuses” category, most notably after 2021. This category includes non-standard affiliations such as special mutual insurance schemes, undefined administrative classifications, and legal incapacitation. Two factors may account for this trend. First, the 2021 reform of the Civil Code, which replaced incapacitation with a rights-based model of supported decision-making [18], may have unintentionally affected how people with severe mental illness are recorded in labour classifications. Second, broader shifts in the social protection system, including the expansion of mutual insurance schemes or non-contributory affiliations, may also explain part of this increase [34]. Taken together, these patterns suggest that the observed decline in disability pensions reflects administrative reallocation rather than genuine improvements in labour participation.

The study period (2018–2023) encompasses the COVID-19 pandemic, which caused severe labour-market disruptions in Spain, including a sharp contraction in employment in 2020 and the widespread use of temporary furlough schemes (ERTEs) that peaked at 3.4 million workers in April 2020 [35]. However, inspection of annual trends does not reveal a clear disruption in employment or disability pension rates attributable to the pandemic in either diagnostic group. This may partly reflect the nature of the BDCAP, which captures administrative affiliation status on December 31st of each year — workers under ERTEs remained classified as employed in the BDCAP, as the database captures administrative affiliation status rather than actual hours worked—and may therefore not fully capture transient pandemic-related fluctuations. This interpretation is consistent with evidence suggesting that, when the pandemic period is excluded, employment trends in Spain show remarkable continuity with pre-pandemic trajectories [36]. The most marked shift observed around 2021 is the growth in the 'other statuses' category, which we attribute primarily to the reform of the Civil Code enacted that year, as discussed above.

Sex-stratified analyses revealed broadly similar patterns of modest increases in employment and declines in disability pensions among men and women, with no significant sex differences across the three diagnostic groups. The only consistent divergence was observed in economic inactivity, which declined more steeply among women with schizophrenia, bipolar disorder, and other diagnoses. These findings are consistent with Nordic register-based studies showing largely parallel employment trajectories for men and women with severe mental illness, although men with bipolar disorder are sometimes reported to have slightly higher employment probabilities than women, while in schizophrenia both sexes often experience persistently low participation [6, 9, 12]. Among individuals with other diagnoses, women also showed greater improvements, particularly in economic inactivity. Together, these results suggest that, while overall trends are comparable across sexes, reductions in inactivity appear to be concentrated among women, possibly reflecting differential transitions within or outside the labour market.

### Strengths and limitations

This study offers several methodological strengths. First, it draws on a large, nationally representative sample of over 26 million individuals, enabling high statistical power and generalizability [37]. Second, it uses validated administrative data from the Spanish Primary Care Clinical Database (BDCAP), which consistently classifies employment status over time. In addition, unlike Nordic studies based on hospital data, our use of primary care records offers a broader view of population health [38]. Third, by combining internal percentages with age-adjusted rates, the study provides complementary insights into both within-group and population-level trends. Fourth, the use of Joinpoint regression allowed us to identify statistically significant trend changes while minimising overfitting. Finally, to our knowledge, this is the first national study to examine long-term employment trajectories in individuals with schizophrenia and bipolar disorder in Spain.

Several limitations should also be acknowledged. First, the observational design does not allow causal inference, and both administrative policies and the exclusion of individuals affiliated with public mutualities who opt for private healthcare—representing around 3.5% of the total Spanish population [39]—may limit representativeness and the interpretation of observed trends. Second, the database does not include important clinical or social variables—such as symptom severity, functional impairment, comorbidities, treatment type, education, income, or family structure—that may influence employment capacity and restrict our ability to adjust for contextual factors. Third, representativeness is further limited by the exclusion of individuals under 20 years of age in employment, which may lead to an underestimation of early labour market participation. Fourth, the “other statuses” category lacks definitional clarity and likely includes a heterogeneous set of affiliations, limiting interpretability. Fifth, employment status reflects only the situation on December 31 of each year and does not capture within-year changes such as temporary or seasonal employment. Sixth, the observation period (2018–2023) is relatively short compared with longitudinal datasets available in other European countries [40]. Despite these limitations, the consistency of our findings across multiple indicators strengthens the validity of the conclusions.

## Conclusion

Employment outcomes remain poor for people with schizophrenia and bipolar disorder in Spain, with men and women showing broadly similar trajectories. The only consistent sex difference was a steeper decline in economic inactivity among women. These findings point to persistent barriers to work for people with severe mental illness and underline the urgency of integrating employment support into mental health policy.

## Data Availability

No datasets were generated or analysed during the current study.
